# The complete chloroplast genome of *Cuscuta pentagona* Engelm

**DOI:** 10.1080/23802359.2018.1467229

**Published:** 2018-04-26

**Authors:** Inkyu Park, Sungyu Yang, Wook Jin Kim, Pureum Noh, Hyun Oh Lee, Byeong Cheol Moon

**Affiliations:** a K-herb Research Center, Korea Institute of Oriental Medicine, Daejeon, Republic of Korea;; b Phyzen Genomics Institute, Seongnam, Republic of Korea

**Keywords:** *Cuscuta pentagona*, parasitic plant, chloroplast genome, Convolvulaceae

## Abstract

*Cuscuta pentagona* is a parasitic plant whose seeds are often mixed with the seeds of medicinal *Cuscuta* species. To facilitate the identification of *C. pentagona* seeds, we generated the complete chloroplast genome sequence of *C. pentagona* Engelm. using the Illumina MiSeq platform. The complete chloroplast genome is 86,380 bp long, with a 50,958 bp LSC region, a 7022 bp SSC region, and two inverted repeat (IRa and IRb) regions comprising 14,200 bp. The chloroplast genome consists of 85 unique genes, 57 protein-coding genes, four ribosomal RNA (rRNA) genes, and 24 transfer RNA (tRNA) genes. Two gene families, NADH oxidoreductases and RNA polymerase-related genes, are absent in this genome. Phylogenetic analysis revealed that *C. pentagona* is closely related to *C. reflexa* and *C. exaltata*, with strong support values.

*Cuscuta pentagona Engelm.*, a member of the Convolvulaceae family, is widely distributed worldwide (Hwang et al. 2013). *Cuscuta pentagona* originated in North America, is a parasitic plant that grows by attaching itself to its host plant and winding around the host via thin, leafless yellow stem with a diameter of approximately 1 mm. This well-known plant parasitizes various hosts and causes extensive crop damage (Alakonya et al. 2012). Although the dried seeds of other *Cuscuta* species are utilized as valuable medicinal plant materials, frequently, these seeds are indiscriminately mixed with *C. pentagona* seeds in the Korean herbal market (KIOM 2016). Thus, we sequenced the chloroplast genome of *C. pentagona* to enable species identification and to protect the herbal medicine market.

Fresh vines were collected from *C. pentagona* plants grown in medicinal plantations in Korea (36°24′56.2″ N 127°23′26.1″ E). The *C. pentagona* plants were given identification numbers, and specimens were registered in the Korean Herbarium of Standard Herbal Resources (Index herbariorum code KIOM) at the Korea Institute of Oriental Medicine (KIOM) under Voucher no. KIOM201701018523. Genomic DNA was extracted from the samples using a DNeasy Plant Maxi kit (Qiagen, Valencia, CA). An Illumina paired-end sequencing library was constructed and generated using the MiSeq platform (Illumina Inc., San Diego, CA). High-quality paired-end reads of approximately 2.1 Gb were obtained from *C. pentagona*. The chloroplast contigs of *C. pentagona* were *de novo* assembled from low-coverage whole-genome sequences.

The complete chloroplast genome of *C. pentagona* is 86,380 bp in length, relatively small compared to other angiosperms (GenBank Accession no. MH121054). The *C. pentagona* chloroplast genome has a typical quadripartite structure consisting of a large single copy (LSC) region of 50,958 bp, a small single copy (SSC) region of 7022 bp, and a pair of inverted repeats (IRa and IRb) comprising 14,200 bp. The GC content of the *C. pentagona* chloroplast genome is 37.9%, with the IR regions having higher GC content (43.2%) than the LSC (36%) and SSC (29.6%) regions. The *C. pentagona* chloroplast genome contains 85 unique genes, including 57protein-coding genes, 24 tRNAs, and 4 rRNAs. Among these, 12 genes were duplicated in the IR regions. The chloroplast genome of *C. pentagona* harbours genes related to photosynthesis but lacks NADH and RNA polymerase genes (Revill et al. 2005). A few tRNA genes are absent in this genome.

To investigate the phylogenetic relationship of *C. pentagona* with other plant species, we aligned 56 protein-coding gene sequences shared by the 20 other taxa. We generated a ML tree containing 13 nodes with 100% bootstrap support values (Figure 1). The phylogenetic relationship is well supported in the Convolvulaceae and Solanaceae families. The ML tree indicates that *C. pentagona* forms a sister relationship with *C. reflexa* and *C. exaltata* in the Convolvulaceae family, with 100% bootstrap support values (Figure 1).

**Figure 1.**
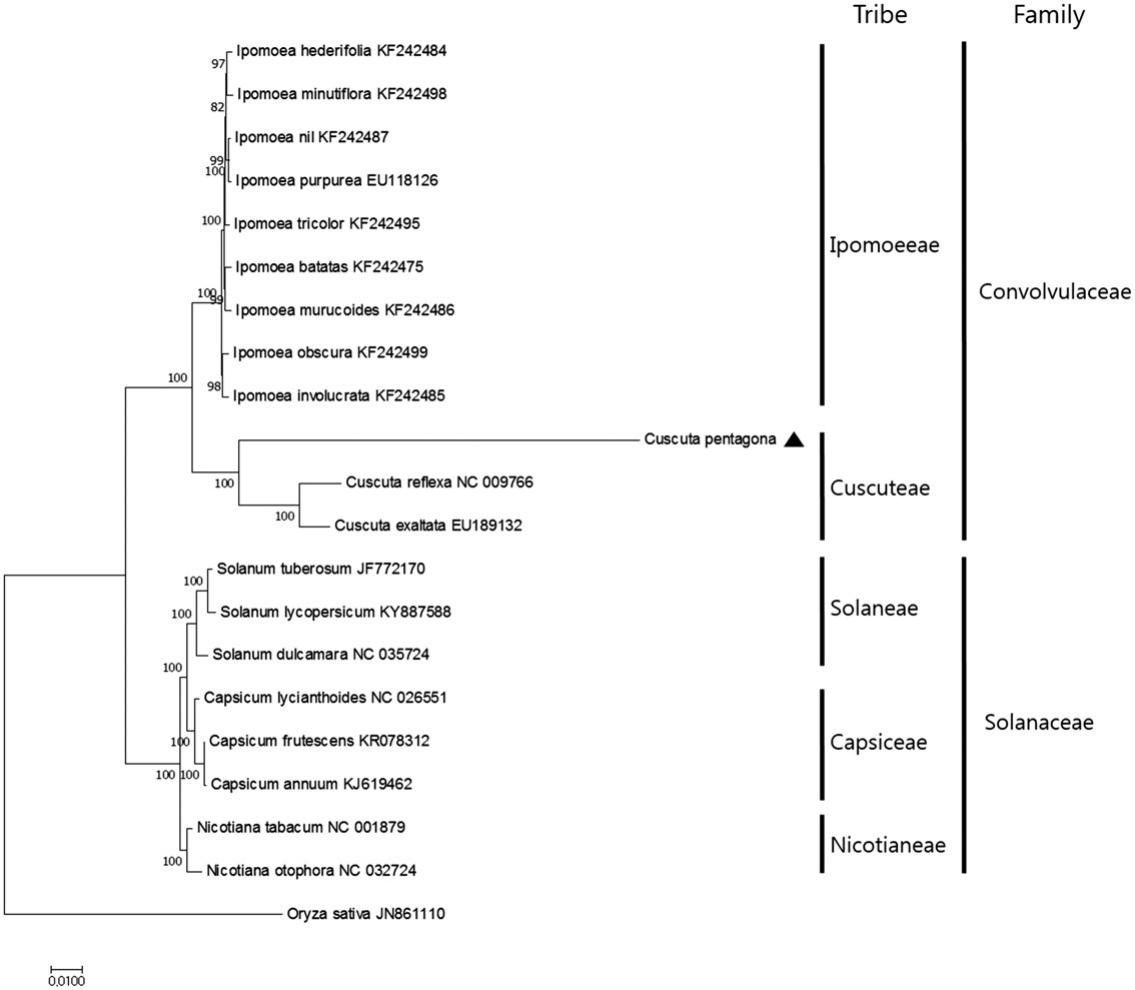
Maximum Likelihood tree based on the chloroplast protein-coding genes of 21 taxa including *C. pentagona* and *Oryza sativa* as outgroup. Fifty-nine protein-coding genes were aligned using MAFFT (http://mafft.cbrc.jp/alignment/server/index.html) and used to generate the Maximum Likelihood phylogenetic tree with MEGA 6.0 (Tamura et al., 2013). The bootstrap support values (>50%) from 1000 replicates are indicated in the nodes.

These findings should facilitate the development of tools that can be used to identify *C. pentagona* to help protect the herbal medicine market.
